# Tracheoesophageal fistula treated with magnetic compression technique in canines

**DOI:** 10.1038/s41598-023-31903-2

**Published:** 2023-03-24

**Authors:** Yixing Li, Miaomiao Zhang, Peinan Liu, Zhixuan Zhang, Hanzhi Zhang, Yi Lyu, Xiaopeng Yan

**Affiliations:** 1 Department of Hepatobiliary Surgery, The First Affiliated Hospital of Xi’an Jiaotong University, 277 West Yanta Road, Xi’an, 710061 Shaanxi China; 2 Department of Thoracic Surgery, The First Affiliated Hospital of Xi’an Jiaotong University, Xi’an, 710061 Shaanxi China; 3 National and Local Joint Engineering Research Center for Precision Surgery & Regenerative Medicine, The First Affiliated Hospital of Xi’an Jiaotong University, 76 West Yanta Road, Xi’an, 710061 Shaanxi China; 4 Qide College, Xi’an Jiaotong University, Xi’an, 710061 Shaanxi China

**Keywords:** Gastrointestinal diseases, Gastrointestinal models, Gynaecological cancer, Biological models, Experimental organisms

## Abstract

There are various surgical methods for tracheoesophageal fistula; however, there is presently no unified standard. Based on the magnetic compression technique, we designed a novel method for the treatment of tracheoesophageal fistula. The purpose of this study was to verify its feasibility in an animal experiment. Six beagle dogs underwent surgical repair after constructing a tracheoesophageal fistula model. After the tracheal and esophageal spaces were freed during the operation, two magnets were used to clamp the fistula. The operation time, intraoperative blood loss, postoperative complications, and wound healing were monitored. Samples were obtained 14 days after the operation, and fistula repair was observed. The tracheoesophageal fistula repair operation was successfully completed for all six beagles. The average operation time was 23.67 ± 4.50 min. The average intraoperative blood loss was less than 10 mL. One dog had a postoperative wound infection, and the rest had no postoperative complications. The wound healed well. In all dogs, after specimen collection, it was observed that the fistula was successfully closed and the mucosal layer was smooth and flat. Histological observation showed that the anastomosis was slightly inflamed, the mucosal layer and surrounding tissues were arranged neatly, and the structure was slightly disordered. Magnetic compression technique can be effectively used to repair tracheoesophageal fistula, shorten the operation time, and simplify the operation procedure, and thus, it has the potential for clinical application.

## Introduction

Tracheoesophageal fistula (TEF) is a pathological passage formed between the trachea and esophagus. It can be caused by congenital malformation, tumors, surgery, and trauma, among other causes. Congenital TEF mostly accompanies esophageal atresia, which occurs in 1/3000–5000 births, and only 4% of congenital TEF cases show an isolated H-type fistula (i.e., TEF without esophageal atresia)^1^. TEF develops in 5–15% of patients with esophageal cancer^2^. Although the incidence of TEF is not high, it has a high mortality rate. Current treatments for TEF include endoscopic clipping, patch repair, stent implantation, and surgical resection. However, endoscopic clipping and stent implantation do not completely cure the condition and are prone to slippage; they may even cause enlargement and recurrence of the fistula^3,4^. Surgical resection includes cervicotomy, thoracotomy, and thoracoscopy^5–7^. Although traditional surgical resection can have a curative effect on resectable TEF, the operation is complicated and time-consuming^4^.

Magnetic compression technique, which is a new anastomosis technology, uses the non-contact force of mutual attraction between magnets to solve various clinical problems^8^. A smooth anastomosis can be formed by pressing the body tissue, and the operation is simpler than traditional manual anastomosis. At present, the magnetic compression technique has been applied in digestive tract reconstruction^8^, vascular anastomosis^9^, and urinary system reconstruction^10^. Therefore, we designed a TEF repair device based on the magnetic compression technique and verified the safety and feasibility of repairing TEF using this device through experiments.

## Material and methods

### Ethical statement

The research protocol and all experimental procedures were strictly in accordance with the Guidelines for the Care and Use of Experimental Animals issued by the Xi'an Jiaotong University Medical Center. This experimental study was approved by the Experimental Ethics Committee of the Xi'an Jiaotong University (Permit number: 2017-773).

### Animals

The study subjects were six beagle dogs (age, 2-5 years; weight, 8-12 kg), all of which comprised the experimental group (this was an exploratory study). The animals were obtained from the Experimental Animal Center, College of Medicine, Xi'an Jiaotong University (Xi'an, China). The animal protocol was designed to minimize pain or discomfort to the animals. The animals were acclimatized to laboratory conditions (23 °C, 12-h/12-h light/dark, 50% humidity, and ad libitum access to food and water) for one week before commencing the experiments. Intramuscular injection of pethidine hydrochloride (1 mg/kg) was administered every 12 h for analgesia for three days after the operation. At the end of the study, all animals were euthanized by barbiturate overdose (intravenous injection, 60 mg/kg pentobarbital sodium) for tissue collection.

### Design of the magnet

According to the structural characteristics of TEF, there is an anatomical gap between the trachea and esophagus. We designed the magnet to be a rectangular parallelepiped measuring 5 cm × 0.5 cm × 0.5 cm. The magnet was made of neodymium–iron–boron (N45) material, and the magnet surface was coated with nickel to improve erosion resistance. The magnetic induction intensity of the magnet surface was 0.35 T. The TEF repair device comprised two magnets of the same size and shape.

### Establishing the animal model

To establish TEF models, our previously reported magnetic compression technique was used on all beagle dogs in this experiment^11^. After the dogs were anesthetized, they were fixed in a supine position. Two cylindrical magnets were placed approximately 3 cm below the glottis in the trachea and in the esophagus using an endoscope. The magnet in the esophagus (diameter, 14 mm; thickness, 5 mm) was slightly larger than the magnet in the trachea (diameter, 10 mm; thickness, 5 mm) to ensure that the magnet can be discharged through the digestive tract after the animal model of TEF is successfully established. The two magnets attracted each other and pressed on the tracheal and esophageal tissues to form an anastomosis, thus establishing a TEF model. The dogs were fed regularly and monitored daily after the operation. Notably, 7–10 days after magnet placement, the dogs coughed after swallowing and showed decreased appetite. X-ray fluoroscopy revealed no magnet in the body. It was found that the magnets were excreted through the digestive tract outside the body. Then, the status of the TEF was studied endoscopically.

### TEF repair

**Figure 1:**
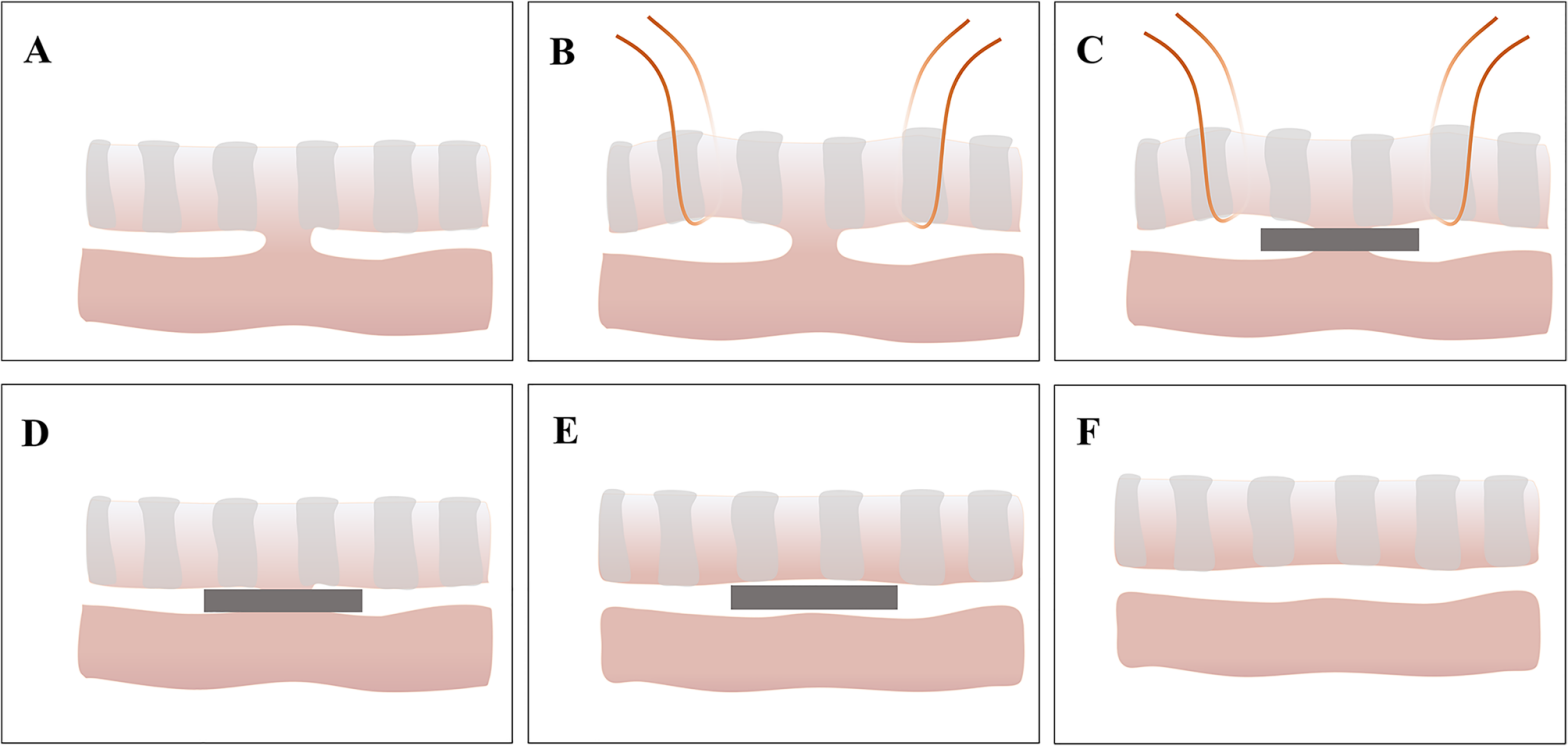
The process of repairing TEF using the magnetic compression technique. (**A**) TEF model formed by the magnetic compression technique. (**B**) The tracheoesophageal space was cleaned and the TEF was exposed. (**C**) The pressing magnet was placed in the tracheoesophageal space. (**D**) The pressing magnet continued to press the fistula in vivo until both sides were healed. (**E**) After the two sides were healed, the pressing magnets were separated from the tissue. (**F**) After removing the pressing magnets, the TEF was repaired.

To proceed with TEF repair, the dogs were anesthetized with 1-mL/kg pentobarbital sodium. Skin preparation, disinfection, and draping were routinely performed, and the dogs were placed in a supine position. The left femoral vein was cannulated to establish venous access, and saline was slowly instilled. Cefazolin sodium (0.5 g) was intravenously injected 30 min before surgery to prevent infection. We made an incision of about 6 cm along the center of the neck and gradually separated the neck tissue, thus exposing and freeing the trachea and esophagus. The tracheoesophageal space was then separated and TEF was exposed. Then, squeezing magnets were placed on both sides of the fistula such that the long axis of the magnet was in the same direction as the trachea and esophagus. After ensuring that no other important tissues were present between the magnets, the magnets were aligned such that they attracted each other, and the fistula was closed. The neck tissue was sutured layer by layer, and the neck incision was closed. The process of repairing TEF using the magnetic compression technique is shown in Fig. 1.

### Postoperative management

All dogs were given intramuscular injection of cefazolin sodium for three consecutive days to prevent infection and pethidine hydrochloride for analgesia for three consecutive days. Experimental animals were given liquid and high-protein diets for the first 5 days after the operation. The healing of the neck wound after the operation was observed daily, and the dogs were monitored for any postoperative complications.

The dogs’ general condition was stable by 14th day postoperatively. Esophagography was first performed before the animals were euthanized, and no contrast agent was developed in the trachea. Then, gastroscopy and bronchoscopy were further performed to confirm fistula closure. Subsequently, all animals were euthanized by a barbiturate overdose (intravenous injection, 60 mg/kg pentobarbital sodium), and the specimens of neck tracheal fistula and esophagus fistula were collected to observe their healing. Then all samples were fixed in 10% formalin. Sections (4–6 µm) were stained with hematoxylin and eosin (HE) and Masson dye for light microscopy.

### Statistical analysis

We used SPSS for descriptive statistical analysis of the data. Measurement data conforming to the normal distribution were expressed as mean ± standard deviation ($\overline{X} \pm s$ ); measurement data with skewed distribution were expressed as M(QR), and count data were expressed as percentages.

## Results

### Surgical conditions and postoperative complications

**Figure 2:**
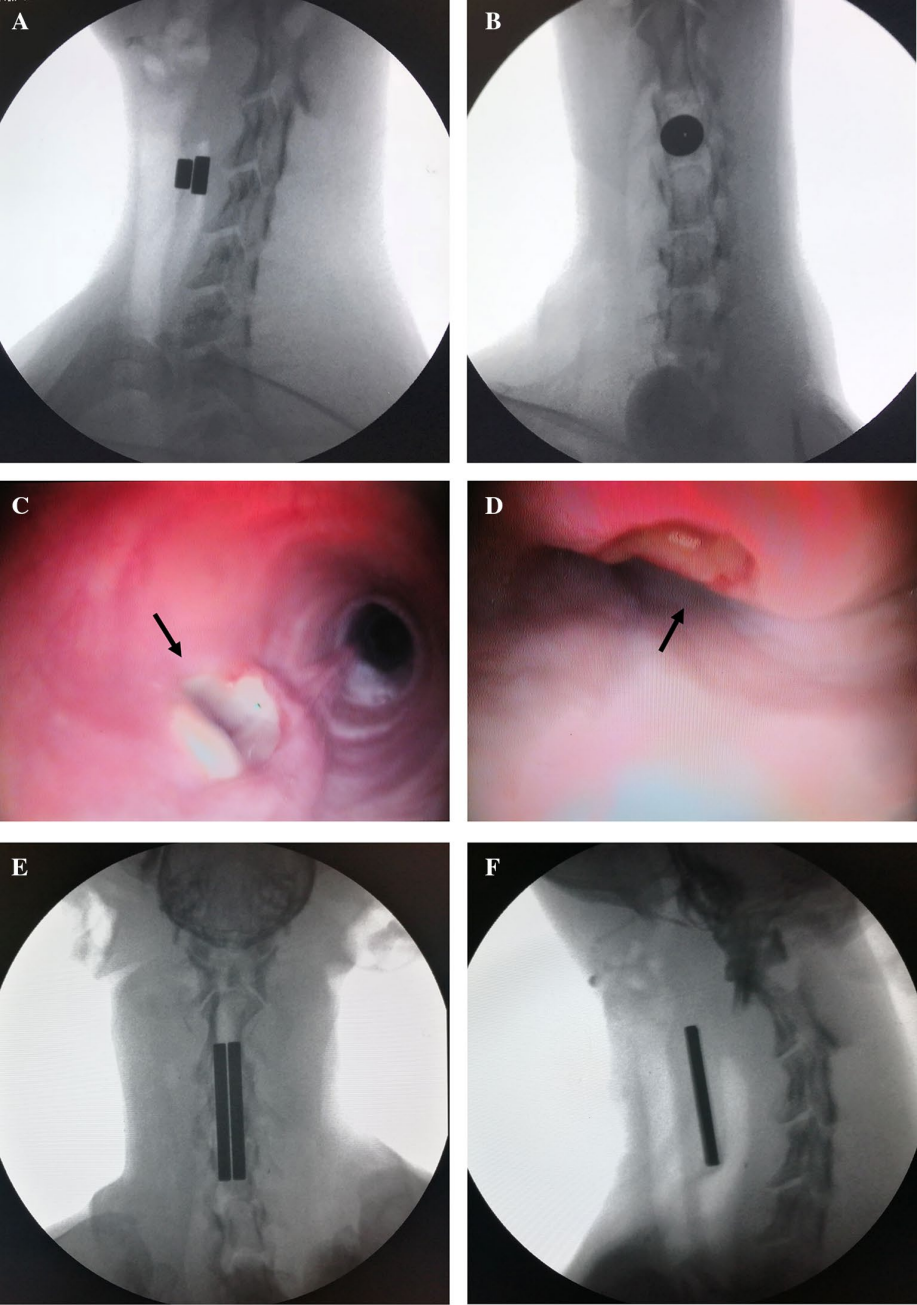
TEF modeling and surgical repair. (**A,B**) Construction of the TEF model—esophagus and trachea magnets attracted each other. (**C**) Bronchoscopy showed TEF in the tracheal membrane. (**D**) Esophagoscopy revealed a fistula in the anterior wall of the esophagus. (**E,F**) The magnets are attracted to each other tightly, squeezing and clamping the TEF without damaging other tissue structures.

All operations were performed by the same team. TEF repairs were successfully completed in all dogs (Fig. 2). The mean operation time was 23.67 ± 4.50 min, and the mean intraoperative blood loss was < 10 mL. Postoperative X-ray fluoroscopy revealed that the magnets were closely attracted to each other, and the TEF was successfully compressed and closed. No vital organs were damaged during the operation, and the magnets did not damage the esophagus wall, tracheal wall, or other important tissues.

Postoperatively, all dogs had poor mental state and poor appetite, which gradually improved after 3–5 days. The anti-infective treatment administered postoperatively continued to improve the dogs’ condition. One of the dogs developed a severe infection in the neck wound, which was treated with a second surgical debridement. The rest of the beagles had no obvious postoperative complications; their neck wound healed well, and the diet returned to normal by 14th day postoperatively. There was no dysphagia or choking after swallowing.

### Observation of in vitro specimens

**Figure 3:**
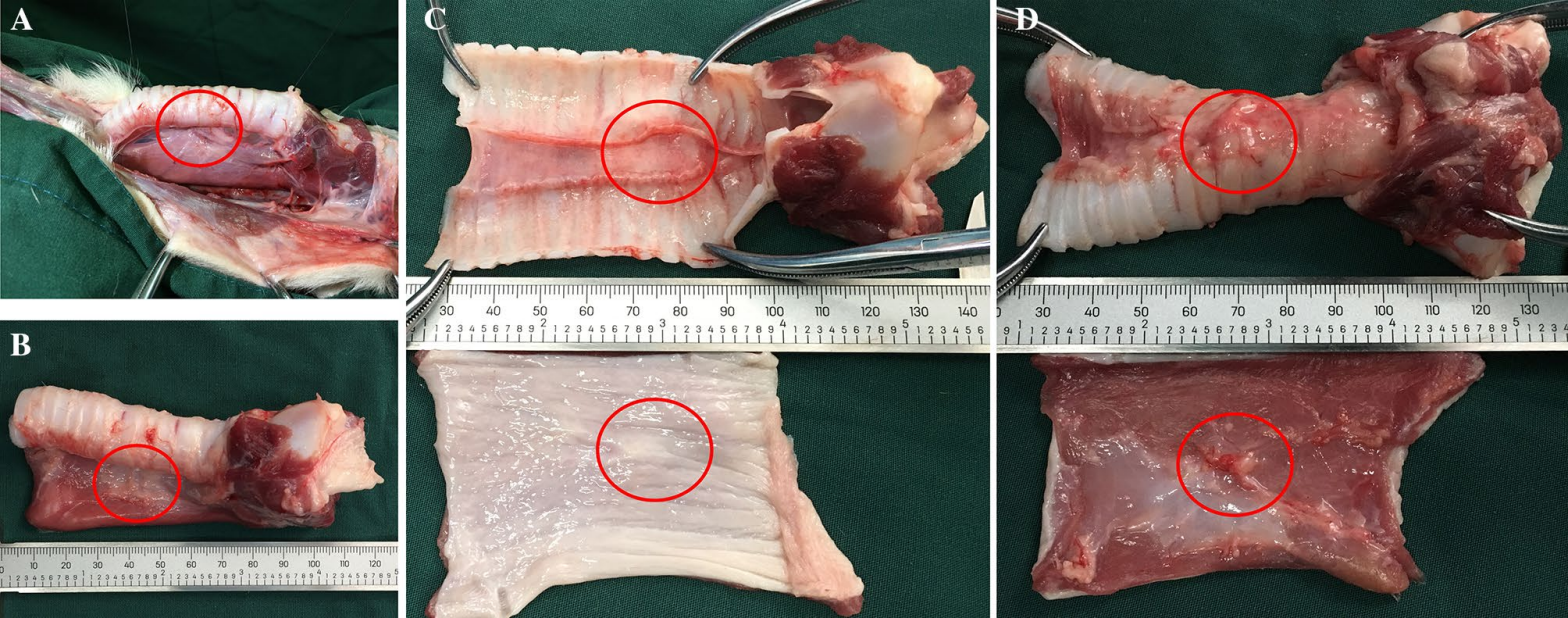
In vitro specimens after TEF repair. (**A**) Esophageal and tracheal space after the removal of pressing magnets. (**B**) Isolated esophagus and trachea specimens. (**C**) The inner side of the tracheal membrane and esophageal mucosa. (**D**) The outer side of the tracheal membrane and esophageal serosa.

Fourteen days after TEF repair, the fistula was healed well, as confirmed by gastroscopy and bronchoscopy, and no anastomotic leakage or anastomotic stenosis was found in any dog (Fig. 3). Esophagography showed no TEF. Subsequently, we obtained the tissue at the anastomosis site of the trachea and esophagus for further observation. During the dissection, we found that there were marginal tissue adhesions around the anastomosis, and the tracheal and esophageal fistulas were successfully closed. The mucosal surfaces of the esophagus and trachea were observed to be smooth and flat, with no significant difference from the surrounding tissue.

### HE staining

**Figure 4:**
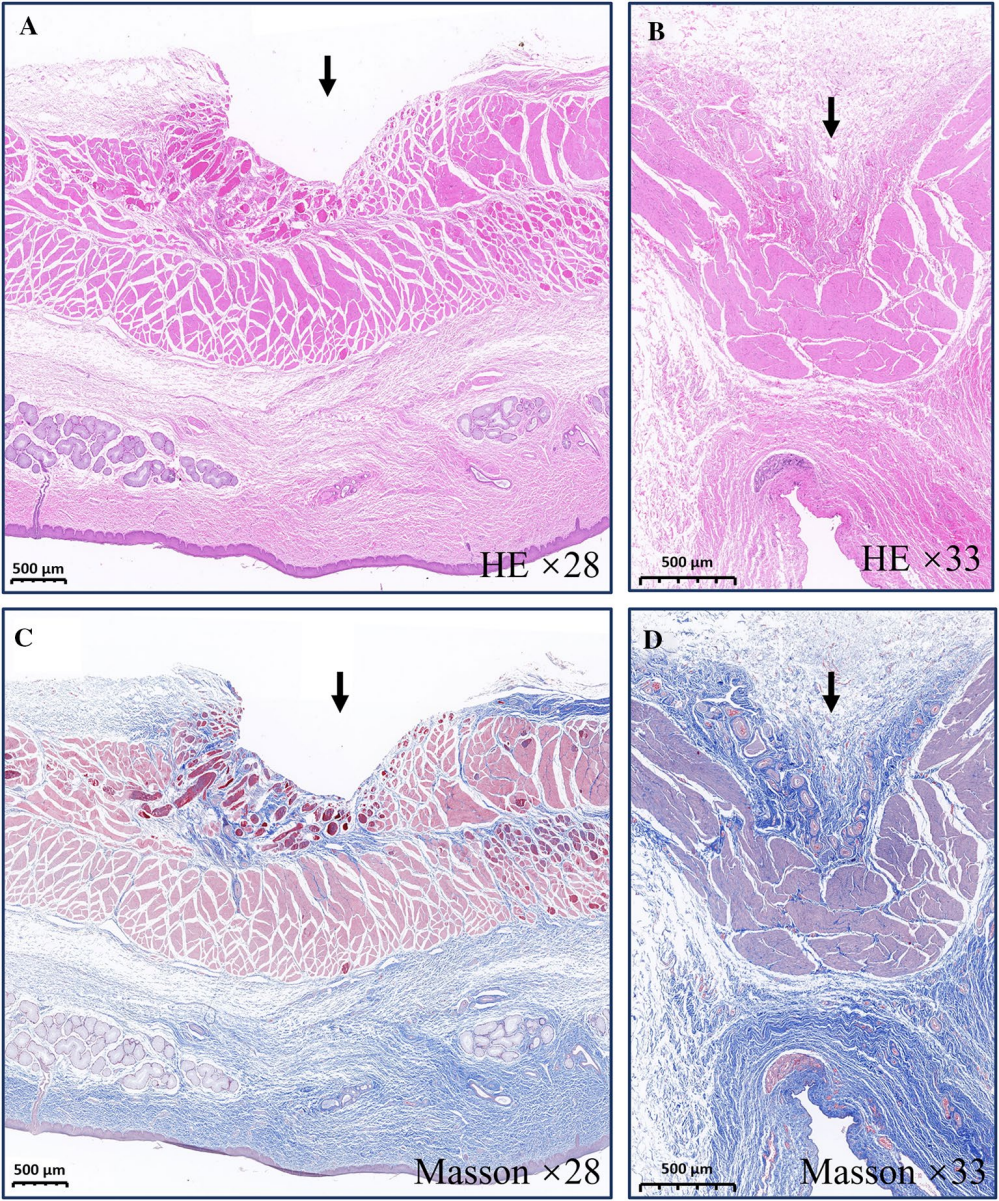
Histological observations. (**A**) HE staining of esophageal specimens (× 28). (**B**) HE staining of the tracheal specimens (× 33). (**C**) Masson’s staining of esophageal specimens (× 28). (**D**) Masson’s staining of the tracheal specimens (× 33).

The local anastomotic tissue of the trachea and esophagus was isolated, embedded in paraffin, and then frozen-sectioned. It was observed under an optical microscope after HE staining (Fig. 4). On both esophageal and tracheal sides, the mucosal layer has good continuity but is slightly thinner than the surrounding tissues. The tissue structure at the anastomosis was slightly disordered.

## Discussion

At present, there are many ways to treat TEF, including endoscopic treatment and surgical resection. The use of titanium forceps to clamp TEF under endoscopy or to block TEF using a stent can effectively relieve the symptoms of patients. The operation trauma is small, and the patients benefit well^12,13^. In particular, for malignant TEF, endoscopic treatment is the first choice to relieve symptoms^14^. However, endoscopic clipping therapy cannot effectively solve the larger TEF, and its surgical success rate is only 50%^15^. Currently, for resectable TEF, surgical resection is the gold standard for clinical treatment, and its success rate can be as high as ~ 90%^16,17^. The most commonly used surgical repair method in the clinic is the three-layer repair method, which involves repairing the tracheal fistula and esophageal fistula and inserting connective tissue into the tracheoesophageal space to cover the fistula to prevent recurrence. However, this method is time-consuming and traumatic and involves many surgical procedures needed to achieve good results. In this procedure, even a part of the trachea needs to be removed, and the clinical efficacy varies.

Based on the characteristics of TEF, we propose the application of the magnetic compression technique to treat TEF. Cope first reported the technique of anastomosis with magnets^18^. Subsequently, the use of this technique gradually expanded, and there have been reports on gastrointestinal anastomosis, vascular anastomosis, and fistula repairs using magnets. Regarding the application of magnets in medical technology centers, our team has also conducted a lot of research, which is currently in early stages. In terms of gastrointestinal reconstruction, the anastomotic stoma formed by the magnetic compression technique is smooth and flat with lesser inflammatory reaction than other surgeries (e.g., open surgery), which can reduce the surgical trauma^19^. Vascular anastomosis with this technique can significantly shorten the operation time. The inferior vena cava anastomosis can be quickly performed using the magnetic compression technique to shorten the operation time of liver transplantation. Compared with manual suture, the operation time using the magnetic compression technique was significantly shorter (27.32 ± 5.12 min vs. 6.25 ± 2.25 min; *P* < 0.001)^9^. In terms of fistula repair, the magnetic compression technique can reportedly achieve ideal results in the treatment of rectovaginal fistula^20^. Our team successfully established a TEF model using a squeezing magnet in the early stage^11^, which shows that the magnet can effectively squeeze the trachea and esophagus. Accordingly, the present study further explored the use of magnetic compression techniques for the treatment of TEF.

The result shows that the magnetic compression technique can effectively treat TEF. After being compressed by the magnet, the tracheal fistula and the esophageal fistula were healed, and the mucosal surface was smooth and flat. There was no obvious stenosis of the esophagus and trachea, and no anastomotic leakage occurred. Through the magnetic compression technique, ischemic necrosis of the clamped tissue was achieved, and the tissue at both ends of the magnet was healed separately. In this experiment, after separating the TEF, the fistula tract was directly squeezed by a magnet without excision or manual suture, which inevitably shortened the operation time, simplified the operation process, and reduced intraoperative blood loss. As the airway was not open, the surgery could be performed without endotracheal intubation. Notably, once the esophageal and tracheal spaces can be clearly separated, the operation with the magnetic compression technique can be performed with relative ease, which we believe will help reduce the difficulty of TEF surgery.

The magnetic compression technique has shown good results in the treatment of TEF, but it has some limitations. First, in this study, the diameter of the fistula in the animal model of TEF was 1 cm, making it a small TEF, and although no anastomotic stenosis occurred after clipping in this study, the efficacy of this method in treating a larger TEF needs to be further explored. Second, the magnets used in this study required a 6-cm neck incision and were placed by manual coaptation. Whether the magnets can be placed in a more minimally invasive way and more easily should be explored in further research. In addition, in this study, as the beagles were euthanized 14 days after the operation, the survival status of the beagles after the removal of the neck magnet was not observed. Whether the magnetic compression technique is effective in preventing postoperative TEF recurrence needs further investigation.

In this study, we focused more on whether magnetic compression technique can achieve TEF repair. It is meaningful to further optimize the design of surgical protocols only after a good TEF repair effect has been proved. Therefore, in this experiment, the magnet was left in the body and removed 14 days after the operation. We were elated with the results as complete healing of the trachea and esophageal fistulas was observed. We hope that this novel treatment method will attract the interest of our colleagues and that their wisdom and knowledge will help further optimize magnet design and surgical procedures. We believe that if this technology can be clinically applied, we will fix the silicone catheter at one end of the magnet and the other end of the catheter leads out of the neck skin. After the repair of the TEF fistula is completed, the silicone catheter can be pulled to remove the two magnets (just like removing the abdominal drainage tube), thus avoiding the need for a second surgical procedure.

## Conclusion

The magnetic compression technique is safe and can repair TEF. It can simplify the operation process, shorten the operation time, and reduce the intraoperative blood loss. No anastomotic stenosis or leakage was noted postoperatively. As a new anastomotic technique, the magnetic compression technique has potential for clinical application in fistula repair.

## Data Availability

The datasets used and analyzed during the current study available from the corresponding author on reasonable request.

## References

[1] Brookes JT, Smith MC, Smith RJ, Bauman NM, Manaligod JM, Sandler AD (2007). H-type congenital tracheoesophageal fistula: University of Iowa experience 1985 to 2005. Ann. Otol. Rhinol. Laryngol..

[2] Ghaffaripour S, Souki FG, Martinez-Lu K, Wakim G (2017). Anesthetic approach for endoscopic repair of acquired tracheoesophageal fistula. Semin. Cardiothorac. Vasc. Anesth..

[3] Sikka K, Singh CA, Agrawal R, Kumar R, Thakar A, Sharma SC (2019). Acquired non-malignant cervical trachea-esophageal fistula: A case series. Indian J. Otolaryngol. Head Neck Surg..

[4] Silon B, Siddiqui AA, Taylor LJ, Arastu S, Soomro A, Adler DG (2017). Endoscopic management of esophagorespiratory fistulas: a multicenter retrospective study of techniques and outcomes. Dig. Dis. Sci..

[5] Conforti A (2016). Cervical repair of congenital tracheoesophageal fistula: Complications lurking!. J. Pediatr. Surg..

[6] Rozeik AE, Elbarbary MM, Saleh AM, Khodary AR, Al-Ekrashy MA (2020). Thoracoscopic versus conventional open repair of tracheoesophageal fistula in neonates: A short-term comparative study. J. Pediatr. Surg..

[7] Slater BJ, Rothenberg SS (2016). Tracheoesophageal fistula. Semin. Pediatr. Surg..

[8] Lu Q (2020). End-to-end vascular anastomosis using a novel magnetic compression device in rabbits: a preliminary study. Sci. Rep..

[9] Cao Z (2020). Fast and effective nonsuture anastomosis of magnetic artificial blood vessel transplantation for caval reconstruction in canines. Ann. Vasc. Surg..

[10] An Y (2023). An experimental study of magnetic compression technique for ureterovesical anastomosis in rabbits. Sci. Rep..

[11] Gao Y, Wu RQ, Lv Y, Yan XP (2019). Novel magnetic compression technique for establishment of a canine model of tracheoesophageal fistula. World J. Gastroenterol..

[12] Armellini E (2015). New endoscopic over-the-scope clip system for treatment of a chronic post-surgical tracheoesophageal fistula. Endoscopy.

[13] Ke M, Wu X, Zeng J (2015). The treatment strategy for tracheoesophageal fistula. J. Thorac. Dis..

[14] Spaander MC (2016). Esophageal stenting for benign and malignant disease: European Society of Gastrointestinal Endoscopy (ESGE) Clinical Guideline. Endoscopy.

[15] Mann C (2019). Surgical treatment of esophagotracheal and esophagobronchial fistulas. Chirurg.

[16] Osho A, Sachdeva U, Wright C, Muniappan A (2018). Surgical management of tracheoesophageal fistula. Ann. Cardiothorac. Surg..

[17] Bibas BJ (2016). Surgical management of benign acquired tracheoesophageal fistulas: A ten-year experience. Ann. Thorac. Surg..

[18] Cope C (1995). Creation of compression gastroenterostomy by means of the oral, percutaneous, or surgical introduction of magnets: Feasibility study in swine. J. Vasc. Interv. Radiol..

[19] Ma F (2019). A novel magnetic compression technique for small intestinal end-to-side anastomosis in rats. J. Pediatr. Surg..

[20] She ZF (2017). Treatment of rectovaginal fistula by magnetic compression. Int. Urogynecol. J..

